# Toll-like receptor 3 as a new marker to detect high risk early stage Non-Small-Cell Lung Cancer patients

**DOI:** 10.1038/s41598-019-50756-2

**Published:** 2019-10-03

**Authors:** Francesca Bianchi, Massimo Milione, Patrizia Casalini, Giovanni Centonze, Valentino M. Le Noci, Chiara Storti, Spyridon Alexiadis, Mauro Truini, Gabriella Sozzi, Ugo Pastorino, Andrea Balsari, Elda Tagliabue, Lucia Sfondrini

**Affiliations:** 1 Molecular Targeting Unit, Department of Research, Fondazione IRCCS Istituto Nazionale dei Tumori di Milano, 20133 Milan, Italy; 2 First Pathology Unit, Department of Pathology and Laboratory Medicine, Fondazione IRCCS Istituto Nazionale dei Tumori di Milano, 20133 Milan, Italy; 3 Tumor Genomics Unit, Department of Research, Fondazione IRCCS Istituto Nazionale dei Tumori di Milano, 20133 Milan, Italy; 4 Thoracic Surgery Unit, Department of Surgery, Fondazione IRCCS Istituto Nazionale dei Tumori di Milano, 20133 Milan, Italy; 5 Pathological Anatomy Unit, ASST Grande Ospedale Metropolitano Niguarda, Piazza dell’Ospedale Maggiore, 3, 20162 Milan, Italy; 6 Università degli Studi di Milano, Dipartimento di Scienze Biomediche per la Salute, via Mangiagalli 31, 20133 Milan, Italy

**Keywords:** Non-small-cell lung cancer, Non-small-cell lung cancer, Tumour biomarkers, Tumour biomarkers, Tumour immunology

## Abstract

Immune and epithelial cells express TLR3, a receptor deputed to respond to microbial signals activating the immune response. The prognostic value of TLR3 in cancer is debated and no data are currently available in NSCLC, for which therapeutic approaches that target the immune system are providing encouraging results. Dissecting the lung immune microenvironment could provide new prognostic markers, especially for early stage NSCLC for which surgery is the only treatment option. In this study we investigated the expression and the prognostic value of TLR3 on both tumor and immune compartments of stage I NSCLCs. In a cohort of 194 NSCLC stage I, TLR3 immunohistochemistry expression on tumor cells predicted a favorable outcome of early stage NSCLC, whereas on the immune cells infiltrating the tumor stroma, TLR3 expression associated with a poor overall survival. Patients with TLR3-positive immune infiltrating cells, but not tumor cells showed a worse prognosis compared with all other patients. The majority of TLR3-expressing immune cells resulted to be macrophages and TLR3 expression associates with PD-1 expression. TLR3 has an opposite prognostic significance when expressed on tumor or immune cells in early stage NCSCL. Analysis of TLR3 in tumor and immune cells can help in identifying high risk stage I patients for which adjuvant treatment would be beneficial.

## Introduction

Toll-like receptors (TLRs) are expressed on immune cells, where they sense microbial invaders and activate downstream signaling cascades that induce the secretion of cytokines and chemokines, culminating in innate and adaptive immune responses^1^. Their expression on immune cells has been widely exploited to promote an antitumor immune response, and various TLR agonists are being examined in preclinical and clinical studies to orchestrate antitumor immunity^2–4^. TLRs are also expressed on epithelial cells, including cancer cells of several histotypes^5^. In addition to promote cytokines secretion, Toll-like receptor 3 (TLR3) activation on cancer cells has been reported to mediate apoptosis in several cancer histotypes, primarily through an extrinsic pathway^6^. TLRs are involve in the regulation of tumor cell growth, but the specific function of each TLRs and the contribution of each member to the inhibition or escalation of cancer are complex. However, few studies have evaluated the functions and the prognostic significance of TLR3 expression separately in tumor and immune cells. TLR3 expression by the tumor parenchyma and the immune cells that infiltrate the tumor in patients with hepatocellular carcinoma (HCC) is associated with greater overall survival (OS)^7^. In contrast, by immunohistochemisty (IHC), tumoral TLR3 expression significantly associates with poor OS in patients with resectable gastric tumors^8^. In breast cancer cases, TLR3 expression in tumor cells by IHC is significantly associated to a high rate of distant metastasis^9^, whereas in neuroblastomas, TLR3 expression on cancer cells by IHC is associated with a favorable prognosis^10^.

In our knowledge, no study has explored TLR3 protein expression in non-small-cell lung cancer (NSCLC). With the idea that the TLR3 expression could represent a new prognostic marker for NSCLC, we focus our attention on expression of TLR3 in patients with stage I NSCLC because for these patents surgery is usually the only proposed treatment. Adjuvant chemotherapy after surgery is offered to a small cohort of patients stage I NSCLC that has a higher risk of relapse based on tumor size and location, and any parameters related with immune features is actually considered to evaluate recurrence risk. In such scenario, we investigated the expression of TLR3 on both cancer and immune compartments in early stage NSCLC, highlighting opposing prognostic functions of this receptor.

## Results

### TLR3 protein expression on tumor cells and immune cells has an opposite prognostic significance in human early-stage NSCLC

The prognostic significance observed *in silico* of TLR3 in stage I NSCLC was investigated by analyzing TLR3 protein expression in 194 human primary NSCLC specimens that were collected at Fondazione IRCCS Istituto Nazionale dei Tumori (INT cohort). All patients had stage I disease, 58% was pT1, 79% was aged ≥60 years, 48% had a body mass index (BMI) ≥25, 89% had a smoking habit, and 74% was male. The tumors were primarily adenocarcinoma (60%) (Table 1).

**Table 1:** Clinical characteristics of NSCLC patients in overall INT cohort and by expression of TLR3 on tumor cells (TLR3-t), stromal immune infiltrate (TLR3-s), and tumor-infiltrating immune cells (TLR3-i).

	Overall cohort	TLR3-t pos^a^	TLR3-t neg	P value^b^	TLR3-s pos^c^	TLR3-s neg	P value^a^	TLR3-i pos^d^	TLR3-i neg	P value^a^
	(N = 194)	(N = 73)	(N = 121)		(N = 110)	(N = 84)		(N = 113)	(N = 81)	
Histology										
Adeno-carcinoma	117 (60%)	39 (53%)	78 (64%)	0.1279	58 (53%)	59 (70%)	0.0135	61 (54%)	56 (69%)	0.0334
others	77 (40%)	34 (47%)	43 (36%)		52 (47%)	25 (30%)		52 (45%)	25 (31%)	
pT										
1, 1a, 1b	112 (58%)	48 (66%)	64 (53%)	0.079	56 (51%)	56 (67%)	0.0277	60 (53%)	52 (64%)	0.1227
2, 2a	82 (42%)	25 (34%)	57 (47%)		54 (49%)	28 (33%)		53 (47%)	29 (36%)	
Age										
<60	40 (21%)	13 (18%)	27 (22%)	0.4523	26 (24%)	14 (17%)	0.2345	28 (25%)	12 (15%)	0.0907
≥60	154 (79%)	60 (82%)	94 (78%)		84 (76%)	70 (83%)		85 (75%)	69 (85%)	
BMI										
<25	100 (52%)	31 (42%)	69 (57%)	0.0493	58 (53%)	42 (50%)	0.7065	58 (51%)	42 (52%)	0.9425
≥25	94 (48%)	42 (58%)	52 (43%)		52 (47%)	42 (50%)		55 (49%)	39 (48%)	
Gender										
Male	143 (74%)	49 (67%)	94 (78%)	0.1054	83 (75%)	60 (71%)	0.5279	81 (72%)	62 (77%)	0.4481
Female	51 (26%)	24 (33%)	27 (22%)		27 (25%)	24 (29%)		32 (20%)	19 (23%)	
Smoker										
Yes	172 (89%)	62 (85%)	110 (91%)	0.2034	99 (90%)	73 (87%)	0.5005	102 (90%)	70 (86%)	0.4048
Never	22 (11%)	11 (15%)	11 (9%)		11 (10%)	11 (13%)		11 (10%)	11 (14%)	

The relationships between categorical variables were examined by chi-square test. Two-sided P values that were below the conventional 5% threshold were considered to be statistically significant. Analyses were conducted using SAS (SAS 9.4 Institute Inc., Cary, NC, USA).

^a^Score of TLR3-t expression: percentage of positive tumor cells ≥3.

^b^Chi-square test.

^c^Score of TLR3-s expression: percentage of positive immune cells ≥1.

^d^Score of TLR3-i expression: percentage of positive immune cells ≥1.

**Figure 1:**
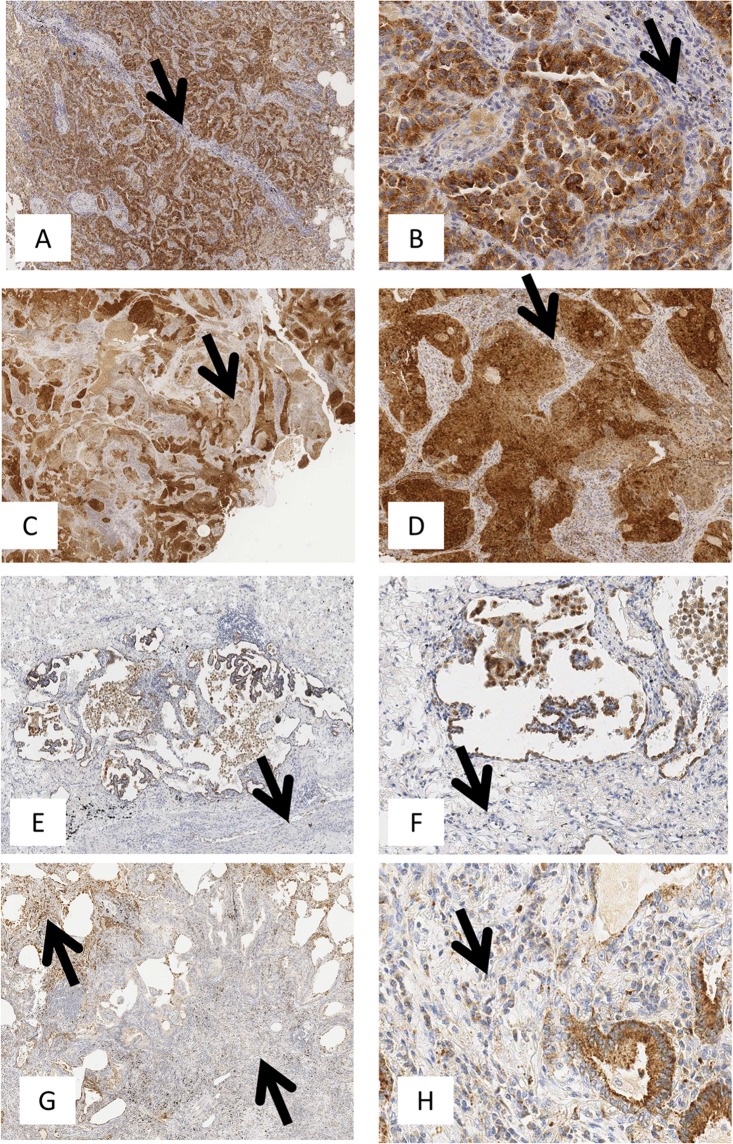
TLR3 immunohistochemical expression in lung adenocarcinoma. TLR3 immunohistochemical staining was performed on FFPE NSCLC tissue as described in Materials and Methods. TLR3 expression was defined in tumor cells and immune cells infiltrating the stroma and in tumor using a semiquantitative method that defined the percentage of positive cells of the total number of cancer or immune cells in the sample (0 = no positive cells; 1 ≤ 25%; 25% < 2 ≤ 50%; 50% < 3 ≤ 75%; 4 > 75%). (**A**–**G**) are images acquired at 100X magnification. (**A**) TLR3 positivity in 95% of tumor cells of the total number of cancer cells (score = 4); (**B**) TLR3 positivity in 65% of tumor cells of the total number of cancer cells (score = 3); (**C**) TLR3 positivity is 30% (score = 2) and 5% of tumor cells of the total number of cancer cells (score = 1); (**D**) Rare TLR3 positivity in neoplastic cells. Neoplastic cells show intense cytoplasmic staining at 200X magnification (**B**–**F**). TLR3 is abundant in neoplastic cells but is expressed in fewer than 25% of non-neoplastic stromal immune cells (**B**,**D**,**F**,**H** arrows; 200X magnification).

IHC performed on FFPE NSCLC specimens using the 40F9.6 mAb^11^ showed TLR3 expression in tumor cells (TLR3-t) and in immune cells infiltrating the stroma (TLR3-s) or the tumor (TLR3-i) (Fig. 1A–H). TLR3 expression was scored using a semiquantitative method based on the percentage of positive cells of the total number of cancer or immune cells in the sample (0 = no positive cells; 1 ≤ 25%; 25% < 2 ≤ 50%; 50% < 3 ≤ 75%; 4 > 75%) (Supplementary Fig. 1).

Distribution of cases by expression pattern of TLR3-t, TLR3-s, or TLR3-i (Supplementary Fig. 1) showed that 123 out of 194 patients expressed TLR3-t (63.4%), enriched in cases with the highest scores—over 50% of cells were positive in these subjects. In the same cohort, 110 patients were positive for TLR3-s (56.7%) and 113 (58.2%) for TLR3-i, with a percentage of TLR3-positive immune cells of ≤25% in most of the cases. Then, these data suggested that the great majority of TLR3 protein in NSCLC arise from tumor cells instead from immune cells.

**Figure 2:**
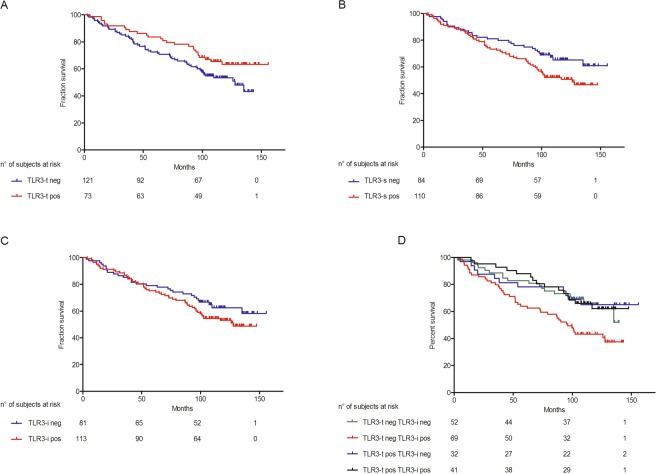
Kaplan-Meier plots of Overall Survival (OS) according to TLR3-t, TLR3-s and TLR3-i immunohistochemistry expression. NSCLC cases were considered positive for TLR3-t expression with a percentage of positive tumor cells >50% and positive for TLR3-s and TLR3-i expression with a percentage of immune cells >0%. Kaplan-Meier plots of OS of 194 NSCLC patients stratified according to TLR3-t, TLR3-s, TLR3-i immunohistochemistry expression and smoking habit are shown. Red line: positive for TLR3 expression; blue line: NSCLC cases negative for TLR3 expression. (**A**) Kaplan-Meier plot of OS of 194 NSCLC patients stratified according to TLR3-t immunohistochemistry expression (p = 0.0503; HR = 0.630; CI = 0.397–1.001; number of events/cases TLR3-t neg: 58/121, TLR3-t pos 26/73); (**B**) Kaplan-Meier plot of OS of 194 NSCLC patients stratified according to TLR3-s immunohistochemistry expression (p = 0.0359; HR = 1.618; CI = 1.032–2.535; number of events/cases TLR3-s neg: 29/84, TLR3-s pos 55/110); (**C**) Kaplan-Meier plot of OS of 194 NSCLC patients stratified according to TLR3-i immunohistochemistry expression (p = 0.1567; HR = 1.380; CI = 0.884–2.155; number of events/cases TLR3-i neg: 30/81, TLR3-i pos 54/113); (**D**) Kaplan-Meier plot of OS of 194 NSCLC patients stratified according to TLR3-t and TLR3-s immunohistochemistry expression (p = 0.0093, Wilcoxon test; number of events/cases TLR3-s pos TLR3-t pos: 15/41, TLR3-s pos TLR3-t neg 41/69, TLR3-s neg TLR3-t pos 11/32, TLR3-s neg TLR3-t neg 18/52). Green line: positive both for TLR3-s and TLR3-t expression; red line: positive for TLR3-s and negative for TLR3-t expression; black line: negative for TLR3-s and positive for TLR3-t expression; blue line: negative both for TLR3-s and TLR3-t expression for TLR3 expression.

Based on the distribution of cases, a cutoff of percentage score of ≥3 (percentage of positive cells ≥ 50%) was used to stratify NSCLC patients to determine the prognostic significance of TLR3-t, and a cutoff of percentage score of ≥1 (percentage of positive cells > 0%) was used to stratify NSCLC patients to determine the prognostic significance of TLR3-s and TLR3-i, in the INT cohort. Of 194 patients, 73 (37.6%) were TLR3-t-positive, based on this cutoff, and by Cox regression univariate analysis, TLR3-t was a positive prognostic factor of OS (p = 0.0503; HR = 0.630; CI = 0.397–1.001) (Table 2; Fig. 2A). Surprisingly TLR3-s resulted a negative prognostic factor of OS (p = 0.0359; HR = 1.618; CI = 1.032–2.535) (Table 2; Fig. 2B), in contrast to what observed for TLR3-t. A trend of association was between TLR3-i and a worse OS (p = 0.1567; HR = 1.380; CI = 0.884–2.155) (Table 2; Fig. 2C).

**Table 2:** Association between TLR3 expression, clinical characteristics, and overall survival (OS) of 194 NSCLC patients.

	Hazard ratio (HR)	95% confidence Iimit (CI)	P value^a^
TLR3-t^b^-positive	0.630	0.397–1.001	0.0503
TLR3-s^c^-positive	1.618	1.032–2.535	0.0359
TLR3-i^c^-positive	1.380	0.884–2.155	0.1567
Histology adenocarcinoma	0.641	0.418–0.981	0.0405
pT 2, 2a	1.408	0.920–2.155	0.1154
Age ≥60	2.223	1.148–4.304	0.0178
BMI ≥25	1.240	0.810–1.899	0.3218
Male gender	3.876	1.940–7.742	0.0001
Smoker	2.644	1.067–2.644	0.0357

OS was defined as the time between the date of surgery and the date of death from any cause or the date of the last follow-up. Univariate survival analysis was carried out by phreg procedure using a Cox regression model and the determination of the statistical significance of all categorical predictors by chi-square test. The effects of explanatory variables on event hazard were quantified by hazard ratios (HR)^33^. All analyses were conducted using SAS (SAS 9.4 Institute Inc., Cary, NC, USA).

^a^Cox regression analysis.

^b^Score of TLR3-t expression: percentage of positive tumor cells ≥3.

^c^Score of TLR3-s and TLR3-i expression: percentage of positive immune cells ≥1.

Considering expression of TLR3 on both tumor and immune cells, NSCLC cases that expressed TLR3-s but not TLR3-t had a significantly worse prognosis compared with all other patients (p = 0.0093; Wilcoxon test) (Fig. 2D).

In the univariate analysis of clinical and pathobiological characteristics, histology, age, smoking habit and gender were significantly associated with OS (P < 0.050; Table 2), whereas none of the other factors in Table 1 had any significant prognostic value. By multivariate Cox survival analysis of all of the covariates that were significant in the univariate analysis, together with male gender (p = 0.0125; HR = 2.558; CI = 1.224–5.348), TLR3-s was a robust, independent prognostic factor (p = 0.0260; HR = 1.683; CI = 1.064–2.662), as was TLR3-t, nearly significantly (p = 0.0511; HR = 0.623; CI = 0.387–1.002) (Supplementary Table 1). No interactions between covariates were significantly associated to the prognosis of NSCLC patients.

The frequencies of the clinical characteristics of NSCLC patients by TLR3-t, TLR3-s, and TLR3-i expression are reported in Table 1. TLR3-t expression was significantly associated with high body mass index (BMI) (p = 0.0493), TLR3-s was significantly associated to adenocarcinoma histology and a high pT (p = 0.0135 and p = 0.0277, respectively), and TLR3-i was significantly associated to adenocarcinoma histology (p = 0.0334); no other pathological parameters associated with TLR3 expression. Moreover, there was no significant association between TLR3 expression on tumor (TLR3-t) and immune cells (TLR3-s) (p = 0.9067), considering the same cutoff of positivity used for OS.

According with smoking habit, tendency towards a good prognosis was observed in smokers expressing TLR3 protein in the tumor cells (p = 0.1461; HR = 0.704; CI = 0.439–1.130) (Supplementary Fig. 2A). Moreover, in smokers a trend towards a worse prognosis and TLR3 protein expression in the tumor infiltrate was observed (p = 0.0856; HR = 1.497; CI = 0.945–2.372) (Supplementary Fig. 2B) and also considering cases expressing TLR3 on immune cells and not on tumor cells (p = 0.0463 Wilcoxon test) (Supplementary Fig. 2C).

Among several immune markers, as CD3, CD4, CD8, HLA-DR and Programmed death 1 protein (PD-1), the only one resulted significantly associated with TLR3 expression on immune cells was PD-1. Indeed, TLR3 expression on pulmonary tumor infiltrate was significantly associated with PD-1 expression (TLR3-s p = 0.0287 Chi square test), and this association was strengthened considering smokers patients (TLR3-s p = 0.0026 Chi square test). The expression of both TLR3 and PD-1 on immune cells infiltrating the tumor stroma in the same cases was strongly associated with the expression of cyclooxygenase-2 (COX-2) on tumor cells (Chi square p value < 0.0001).

**Figure 3:**
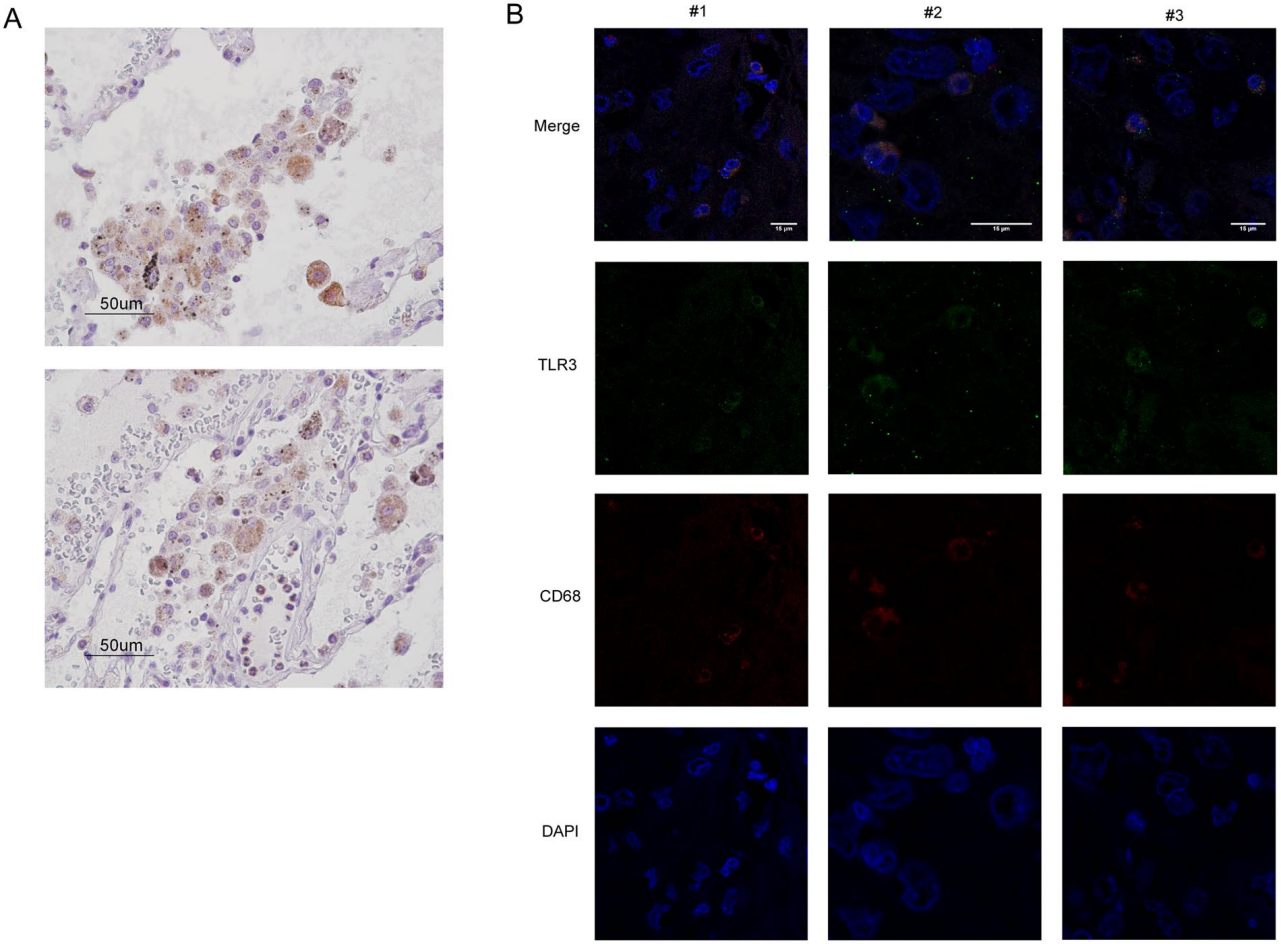
TLR3 expression on immune cells identifies macrophages in NSCLC. (**A**) NSCLC FFPE specimens were stained with anti-TLR3, and TLR3-expressing immune cells were morphologically identified to be macrophages by the pathologist. Two representative images are shown. (**B**) NSCLC FFPE specimens were stained with DAPI (blue), anti-CD68 (red), and anti-TLR3 (green) for co-immunofluorescence assay, and images were acquired on a confocal microscope. Images of 3 representative areas are shown (#1–3). Co-localization of CD68 with TLR3 (yellow; white arrows) confirmed that the stained immune cells were macrophages—not T or B lymphocytes.

On pathological examination of NSCLC specimens, morphologically, the immune cells that expressed TLR3 were nearly exclusively macrophages, harboring few granulocytes (Fig. 3A). By immunofluorescence assay, colocalization of TLR3 with CD68, a marker of the macrophage lineage, confirmed that the TLR3-expressing immune cells were macrophages (Fig. 3B). To investigate the functional phenotype of TLR3-expressing macrophages, we analyzed by IHC analysis the expression of CD163, marker of M2 macrophages reported to function in immune suppression and also tumor progression, on a portion of NSCLC specimens whose material were available. We observed a tendency toward significance of correlation between CD163 and TLR3 expression on immune cells (Pearson r p value 0,0896; n = 16).

These data indicate that TLR3 expression on immune cells in the lung can be detrimental, and that TLR3-positive tumor macrophages might reveal an immunosuppressive context that contribute to sustain the progression of early stage NSCLC.

### TLR3 mRNA expression is associated with a good prognosis in early-stage NSCLC

**Figure 4:**
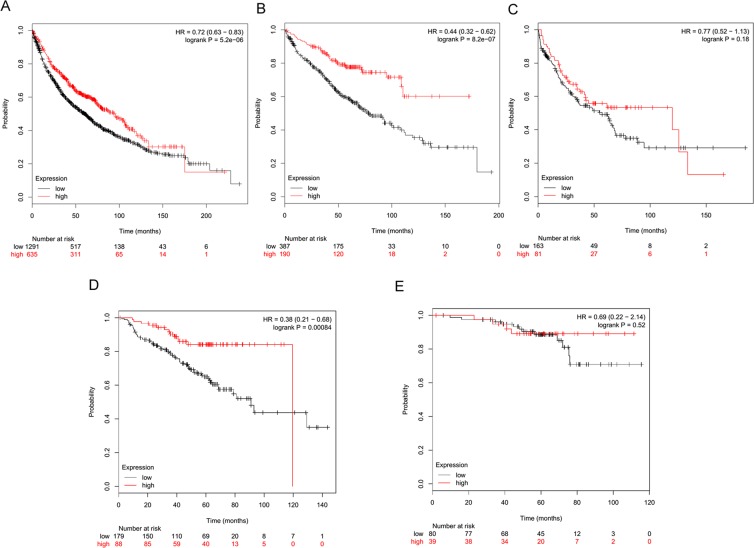
TLR3 mRNA expression is associated with a good prognosis in early-stage NSCLC. TLR3 expression was examined in the KM-Plotter public gene expression NSCLC datasets^12^. NSCLC patients were stratified by tertiles with regard to TLR3 mRNA expression (probe ID 206271_at). Red line: high TLR3 expression; black line: low TLR3 expression. (**A**) OS probability of patients by TLR3 mRNA level, n = 1926. (**B**) OS probability of patients by TLR3 mRNA in only stage I NSCLC cases, n = 577. (**C**) OS probability of patients by TLR3 mRNA in only stage II NSCLC cases, n = 244. (**D**) OS probability of patients by TLR3 mRNA in only stage I smokers NSCLC cases, n = 267. (**E**) OS probability of patients by TLR3 mRNA in only stage I no smokers NSCLC cases, n = 99.

TLR3 expression was validated in the KM-Plotter public NSCLC gene expression dataset^12^. Kaplan-Meier analysis of cases for whom survival information was available (n = 1926) was performed, dividing NSCLC cases into tertiles. Specifically, based on the results of our IHC analysis of TLR3 protein in 194 NSCLC cohort revealing one-third of the case study (37.6%) with a percentage of TLR3 positive tumor cells ≥50%, we analyzed NSCLC cohort in KM-Plotter database dividing cases into tertiles, according to levels of TLR3 mRNA expression, and the highest quantile was considered to be TLR3-positive similarly to what defined for IHC. Patients with TLR3-positive tumors had a better OS than those with TLR3-negative tumors (p < 0.01; HR = 0.72; n = 1926) (Fig. 4A). By multivariate analysis of TLR3 expression and available covariates (histology, gender, and smoking history), TLR3 still was a strong independent prognostic factor of OS (n = 649; p < 0.01; HR = 0.43). This is in accordance with what previous observed in IHC analysis, since distribution of cases by expression pattern of TLR3 suggested that the great majority of TLR3 protein in NSCLC arise from tumor cells instead from immune cells.

Of note, the prognostic value of TLR3 expression resulted more pronounced considering stage I NSCLC cases only (p < 0.01; HR = 0.44; n = 577) (Fig. 4B). Conversely, in more advanced NSCLC, no association between TLR3 and OS was observed, as shown in stage II NSCLC (p = 0.18; HR = 0.77; n = 244) (Fig. 4C). Also multivariate analysis confirmed the prognostic significance of TLR3 as seen when the analysis was restricted to stage I NSCLC (n = 383; p < 0.01; HR = 0.34).

In agreement with what observed in IHC analysis, considering smoking habit, TLR3 was strongly significantly associated with good OS in smokers stage I NSCLC (p < 0.01; HR = 0.38; n = 267) (Fig. 4D) and not associated in no smokers (p = 0.52; HR = 0.69; n = 119) (Fig. 4E). These data suggest that TLR3 expression could be a strong marker of good prognosis in stage I, NSCLC smokers.

## Discussion

In our cohort of 194 stage I NSCLCs investigated by IHC, we observed that TLR3 protein expression on tumor cells is associated with a good OS. Our data are strengthened by the use of a validated antibody for IHC analysis of human TLR3 expression^11^ and by the large stage I NSCLC cohort that was analyzed. Over 50% of subjects remained alive at the 150-months follow-up, and half of the survivors expressed TLR3-t. Noteworthy, despite the analyzed cohort mainly consists of subjects with a tendency to a good prognosis, the prognostic value of TLR3-t remained significant in the multivariate analysis, suggesting that danger exogenous signals to which lung is constantly exposed may sustain TLR3-t activation and apoptosis in tumor cells. Consistent with this speculation, TLR3 was reported to induce apoptosis in cancer cells that were treated *in vitro* with a synthetic TLR3 agonist^13,14^.

In gene expression-based datasets of NSCLC, we found that TLR3 significantly predicts a good prognosis *in silico*, similar to what was recently reported by Bauer and colleagues^15^.

Bauer and colleagues calculated the log-rank P stratifying by the median values for TLR3 gene expression as the threshold for high and low expression. Here, we considered a different cut-off to stratify patients, closely to what we defined for IHC.

Based on our findings, we speculate favorable prognostic value of TLR3 mRNA depends on the abundance of mRNA of tumor versus tumor-infiltrating immune cells origin in a whole-transcriptome analysis. Indeed, in the distribution of TLR3 in our NSCLC INT cohort by IHC, 60% of TLR3-expressing cases contained over 50% of positive tumor cells; in contrast, TLR3-s-positive cases harbored no more than 25% of positive immune cells, indicating that more TLR3 mRNA originated from the tumor compared with the stroma. Thus, the prognostic value of TLR3 mRNA in the *in silico* NSCLC datasets presumably reflects the favorable role of tumoral TLR3.

Investigating TLR3 prognostic role according with the NSCLC stages, we observed that the significant association with OS occurs in stage I tumors, whereas no association was found in more advanced NSCLC, differently to what reported by Bauer and colleagues. No association observed between TLR3 mRNA expression and better prognosis in stage II NSCLC patients likely reflects a resistance to TLR3-mediated apoptosis acquired by tumor cells during tumor progression.

Besides on tumor cells, TLR3 protein resulted to be expressed on immune cells in nearly half of the analyzed NSCLC cases, considering immune cells that infiltrated the stroma or tumor cell islets. Our data indicate that most of the TLR3-expressing immune cells were macrophages and, in contrast to that observed in tumor cells, stromal tumor-infiltrating immune cells that expressed TLR3 (TLR3-s) were strongly significantly associated with a poor prognosis in NSCLC in the univariate and multivariate analyses.

TLR-3 is expressed by sentinel cells of the innate immune system, such as dendritic cells and macrophages, where it senses viral and host-derived nucleic acids and initiates inflammatory pathways, and by non immune cells, including epithelial cells, fibroblasts, and endothelial cells^16,17^. Lung is constantly exposed to exogenous TLR3 ligands and it is plausible that in the lung microenvironment TLR3 is constantly activated. TLR3 activation on immune cells can boost the inflammatory immune mileau towards a tumor supporting microenvironment. Consistently, comparing immune infiltrate of NSCLC according with TLR3 expression, TLR3-s was found significantly associated with PD-1 on immune cells, whose interaction with its ligand PD-L1 on the tumor cells reduces function signals to prevent the immune system from attacking the tumor cells^18,19^, suggesting that TLR3 expression on immune cells infiltrating the tumor stroma might contribute to sustain an irresponsive immune environment. To support the hypothesis of an association of these markers with an immunosuppressive status, the expression of both TLR3 and PD-1 on immune cells infiltrating the tumor stroma was strongly associated with the expression on tumor cells of COX-2, one of the major player of immunosuppression on both innate and adaptive response in the tumor microenvironment^20^, suggesting that tumor inflammatory microenvironment in these cases can recruit/drive immune cells with immunosuppressive features.

Accordingly, considering smoking habit, TLR3 prognostic significance was observed. 80% of NSCLC patients are smokers and cigarette smoke (CS) long-term exposure to the lung can result in chronic inflammation that generates an inflammatory microenvironment driving lung tumor progression^21^. CS exposure, in addition to inducing dysplastic epithelial changes, hyperactivates local innate and adaptive immunity^22^. Thus, TLR3 activation by dsRNA that is released by CS-damaged cells can increase inflammation at tumor site.

Although TLR3-s and TLR3-i were positively associated each other, only TLR3-s associated significantly with a poor prognosis, whereas intratumoral infiltrating immune cells that expressed TLR3 (TLR3-i) was weakly associated with a poor OS.

The lack of significance for TLR3-i could be explained by the lower number of total immune cells in the tumor versus the surrounding stroma,^23,24^. A recent meta-analysis of 29 small and large studies of the prognostic value of various tumor-infiltrating immune cells in lung cancer indicated that stromal assessments of tumor-infiltrating immune cells have a superior prognostic impact compared with intratumoral assessments^25^. Similar observations of the power of tumor-infiltrating immune cells as a prognostic marker, considering intratumoral and peritumoral cells, have been reported for breast cancer^26^.

To estimate patient outcomes, many prognostic factors are available for lung cancers, including both well-known host-related features - such as patient age, gender, smoking status, smoking cessation - and tumor-related factors – such as tumor stage and grade and histology.

There are plenty of publications about biological markers not measured routinely in clinical practice, however, most of these factors are not reproducible and/or their prognostic independent value is not proven. Here, TLR3 results a robust and independent prognostic factor in a multivariate analysis together with other several clinical characteristics (histology, size, age, BMI, gender, smoke habits), and consideration of TLR3 expression both on tumor and immune cells, by using routinely un-expensive IHC approach, can help to identify high risk patients eligible for an adjuvant treatment among NSCLC stage I patients.

The prognostic significance of TLR3 expression in cancer it has been investigated in others cancer histotypes, mainly by qPCR. In patients with hepatocellular carcinoma (HCC), neuroblastomas or esophageal squamous cell carcinoma, TLR3 was associated with greater survival^7,10,27^, while its expression was significantly associated with poor overall survival in patients with resectable gastric tumors or breast cancer^8,9^. Our data indicate that the function of TLR3 on cancer cells must be distinguished from that on immune cells and then the discrepancy about the TLR3 prognostic significance among various cancer histotypes could be ascribed by the variability in immune cells infiltrating the tumor and in the level of TLR3 expression.

In conclusion, our findings indicate that TLR3 is expressed on cancer cells and on immune cells of the majority of early-stage NSCLC patients with an opposite prognostic significance and highlight the value of measuring the levels of this receptor, both in tumor cells and infiltrating immune cells, to identify a subgroup of high risk patients, among early stage NSCLC, that expressing TLR3-s and not TLR3-t had a worse prognosis and for which adjuvant treatment would be beneficial.

## Materials and Methods

### TLR3 expression in NSCLC by gene expression microarray

TLR3 expression was assessed in a large meta-analysis of NSCLC datasets on an Affymetrix platform^12,28^. The online KM-Plotter database, which to date includes information on 22,277 genes and their influence on survival in 1926 NSCLC patients, was used for the survival analysis. NSCLC patients were stratified by tertiles of TLR3 mRNA expression (probe ID 206271_at). The OS of patients by TLR3 mRNA expression was calculated for the entire cohort (n = 1926) and considering only stage I NSCLC cases (n = 577).

### Patients

Samples from 194 NSCLC patients who had been diagnosed between 2003 and 2007 at our institute (Fondazione IRCCS Istituto Nazionale dei Tumori) were selected, based on stage (stage I) and availability of follow-up data. Institutional approval from the Independent Ethics Committee of Fondazione IRCCS Istituto Nazionale Tumori was obtained for the conduct of this study. Patients agreed to the use of their own samples with informed consent. All procedures were in accordance with the 1975 Helsinki Declaration. The median follow-up time of the cohort of 194 patients was 105.7 months.

### Immunohistochemical (IHC) analysis of FFPE NSCLC specimens

See Supplementary Table 2 for antibodies sources and dilutions. TLR3 was analyzed by IHC on 2.5/3-µm formalin-fixed, paraffin-embedded (FFPE) tumor sections, using the anti-TLR3, which was developed by Salaun *et al*.^11^ and kindly provided by Innate Pharma (Innate Pharma, Marseille France). Briefly, antigen retrieval was performed by heating the slides for 30 min at 98 °C in Dako PT-link, EnVision™ FLEX Target Retrieval Solution High pH. Immunoreactions were visualized using a commercially available detection kit (EnVision™ FLEX+, Dako, Denmark) on an automated immunostainer (Dako Autostainer System), and the sections were counterstained with hematoxylin.

To minimize assessment variability, IHC results for each protein were rendered semi-quantitatively by adopting a scoring system taking into account staining marker extent (% positive cells). The expression was defined as follows: 0 = no positive cells; 1 ≤ 25%; 25% < 2 ≤ 50%; 50% < 3 ≤ 75%; 4 > 75%^29,30^.

The reactivity of monoclonal anti-TLR3 in the IHC analysis of FFPE NSCLC specimens was scored as positive for tumor cells when ≥50% of them showed membrane and cytoplasmic staining TLR3 and positive for immune cells when >0% of them showed membrane and cytoplasmic staining TLR3. The cutoff was chosen, based on the distribution of the percentages of TLR3-positive cells in the IHC assay in each tumor section. The same cut-off was used to score PD-1 positivity on immune cells.

We considered as tumor-infiltrating the immune cells in tumor nests having a direct cell-to-cell contact, while as immune cells infiltrating the stroma those that were located dispersed in the stroma between the cancer cells and not directly in contact with carcinoma cells^31,32^. Neoplastic and non neoplastic cells (immune cells) evaluation was driven by the continuous comparison between TLR3, or PD-1, stained specimens and parallel specimens stained for the following immune cells specific antibodies (CD3, CD4, CD8) and for the specific tumoral markers such as general epithelial ones (Cytokeratin AE1-AE3, Cytokeratin 7, EMA, Cytokeratin 8/18) and lung epithelium specific ones (TTF1, p40 and Napsin).

According to those rules, we were able to evaluate TLR3, or PD-1, separately, first in neoplastic compartment (stained by at least by one of the aforementioned lung specific markers but negative for immune cells markers) and then in non neoplastic immune cells, that resulted positive for immune cells markers but negative for epithelial markers.

The specificity of each antibody was verified using appropriate positive and negative controls. To exclude antibody unspecific binding, we replaced the primary antibody with a non-related mouse immunoglobulin at comparable dilutions or using normal serum alone. Images were acquired on an Aperio ScanscopeXT® (Leica Biosystems Aperio) at 40x and 400X magnifications.

### Statistical analysis

Clinical data were accessed when available. The relationships between categorical variables were examined by chi-square test. Two-sided P values that were below the conventional 5% threshold were considered to be statistically significant. OS was defined as the time between the date of surgery and the date of death from any cause or the date of the last follow-up. OS curves were drawn by life-table method, and the statistical significance was tested by log-rank test. After the univariate analysis by phreg procedure using a Cox regression model and the determination of the statistical significance of all categorical predictors by chi-square test, all predictors with p-values < 0.1 were retained for the multivariate analysis. Multivariate survival analysis was carried out using Cox proportional hazards regression models, and the effects of explanatory variables on event hazard were quantified by hazard ratios (HR)^33^. All analyses were conducted using SAS (SAS 9.4 Institute Inc., Cary, NC, USA).

### Fluorescence confocal microscopy analysis of FFPE NSCLC specimens

CD68 and TLR3 immunofluorescence on FFPE were processed and acquired as described^34^. Briefly, monoclonal mouse anti-human CD68 Clone PG-M1 (Dako Omnis) and anti-TLR3 clone 40F9.6^11^, were labeled with Zenon™ Alexa Fluor™ 546 Mouse IgG1 Labeling Kit and Zenon™ and Alexa Fluor™ 488 Mouse IgG1 Labeling Kit (Thermo Fisher Scientific), respectively. Imaging was performed using a confocal laser-scanning microscope Leica TCS SP8 X (Leica Microsystems), equipped with a pulsed super continuum White Light Laser (470–670 nm; 1 nm tuning step size). The fluorochromes were excited by a continuous wave 405 nm diode laser and a pulsed super continuum White Light Laser (470–670 nm; 1 nm tuning step size). In particular, AlexaFluor-488 was excited selecting 488 nm-laser line and detected from 499 nm to 547 nm and AlexaFluor-546 was excited with 555 nm-laser line and detected from 562 to 674 nm. Images were acquired in the scan format 1024 × 1024 pixel using a HC PL APO 40X/1.30 CS2 oil immersion objective and a pinhole set to 1 Airy unit. Data were analyzed using the software Leica LASX rel.1.1 (Leica Microsystems).

### Ethical approval

The tissue specimens in this study were collected during standard surgical procedures at Fondazione IRCCS Istituto Nazionale dei Tumori di Milano. Institutional approval from the Independent Ethics Committee of Fondazione IRCCS Istituto Nazionale Tumori was obtained for the conduct of this study. Patients agreed to the use of their own samples with informed consent. All data were analyzed anonymously, and all experiments complied with the 1975 Declaration of Helsinki.

## Supplementary information

Supplementary Files

## Data Availability

The dataset analyzed during the current study is available from the corresponding author on reasonable request.
