# Supplementary material for: Toll-like receptor 3 as a new marker to detect high risk early stage Non-Small-Cell Lung Cancer patients
Source: Sci Rep. 2019 Oct 3;9:14288. doi: 10.1038/s41598-019-50756-2 (PMC6776648; doi:10.1038/s41598-019-50756-2)
Supplement: Supplementary file 1 — Supplementary Files [file 41598_2019_50756_MOESM1_ESM.pdf]

Toll-like receptor 3 as a new marker to detect high risk early stage Non-Small-Cell Lung Cancer patients

Francesca Bianchi, Massimo Milione, Patrizia Casalini, Giovanni Centonze, Valentino M. Le Noci, Chiara Storti, Spyridon Alexiadis, Mauro Truini, Gabriella Sozzi, Ugo Pastorino, Andrea Balsari, Elda Tagliabue and Lucia Sfondrini.

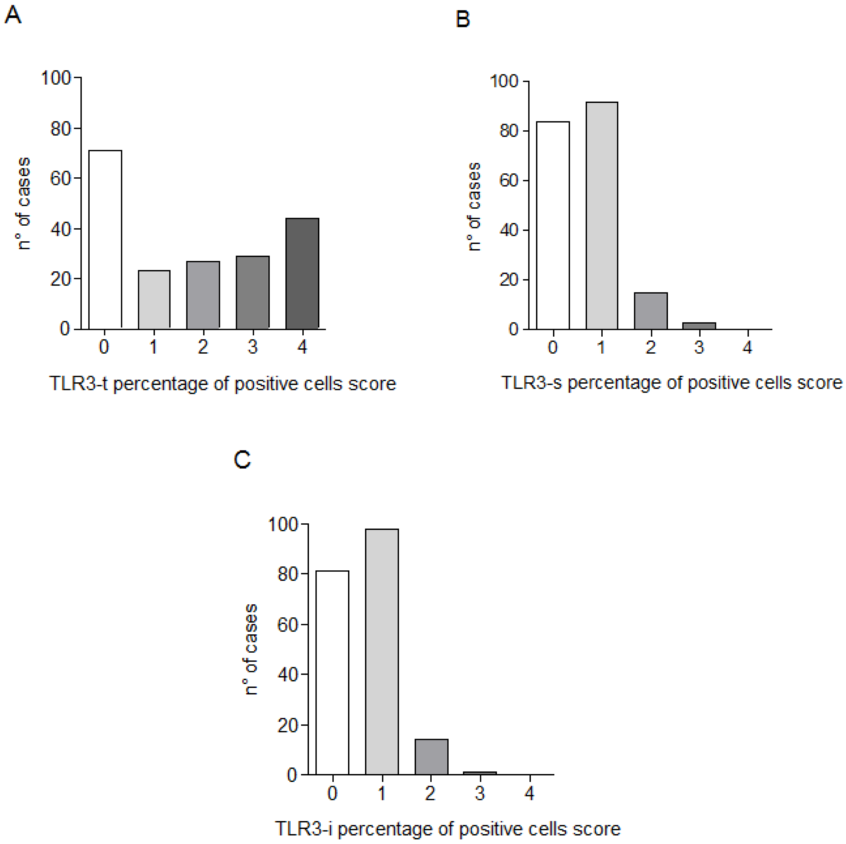

Supplementary Fig. 1. Distribution of 194 NSCLC cases according to the percentage scores of TLR3-t, TLR3-s, TLR3-i. TLR3 expression on tumor cells (TLR3-t) (A), immune cells infiltrating the stroma (TLR3-s) (B) and immune cells infiltrating the tumor (TLR3-i) (C) was evaluated by the pathologist as percentage of positive cells out of the total number of immune cells within the sample (0; 1 ≤25%; 25 < 2 ≤50%; 50 < 3 ≤75%; 4 > 75%).

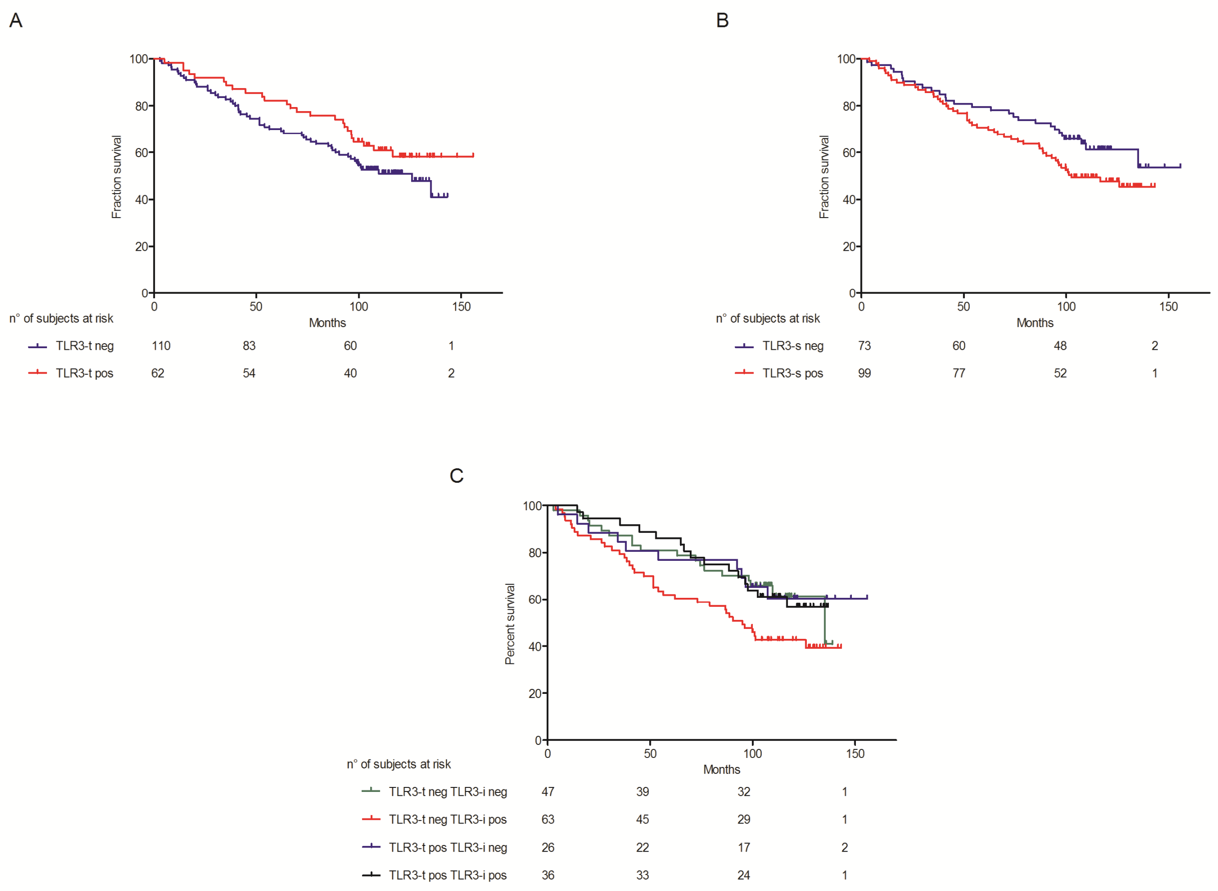

Supplementary Fig. 2. Kaplan-Meier plots of Overall Survival (OS) according to TLR3-t and TLR3-s immunohistochemistry expression and smoking habit. NSCLC cases were considered positive for TLR3-t expression with a percentage of positive tumor cells >50% and positive for TLR3-s expression with a percentage of immune cells >0%. Kaplan-Meier plots of Overall Survival (OS) of 172 NSCLC patients stratified according to TLR3-t and TLR3-s immunohistochemistry expression and smoking habit are shown. Red line: positive for TLR3 expression; blue line: NSCLC cases negative for TLR3 expression. A) Kaplan-Meier plot of OS of 172 NSCLC smokers patients stratified according to TLR3-t immunohistochemistry expression; B) Kaplan-Meier plot of OS of 172 NSCLC smokers patients stratified according to TLR3-s immunohistochemistry expression; C) Kaplan-Meier plot of OS of 172 NSCLC smokers patients stratified according to TLR3-t and TLR3-s immunohistochemistry expression. Green line: positive both for TLR3-s and TLR3-t expression; red line: positive for TLR3-s and negative for TLR3-t expression; black line: negative for TLR3-s and positive for TLR3-t expression; blue line: negative both for TLR3-s and TLR3-t expression for TLR3 expression.

Supplementary Table 1. Multivariate proportional hazards analysis of overall survival (OS) in 194 NSCLC patients.

	Hazard ratio (HR)	95% Confidence limit (CI)	P value[a]
TLR3-t[b] positive	0.623	0.387 – 1.002	0.0511
TLR3-s[c] positive	1.683	1.064 – 2.662	0.0260
Age ≥60	2.021	1.012 – 4.036	0.0461
Histology adenocarcinoma	0.777	0.501 – 1.206	0.2608
Male gender	2.558	1.224 – 5.348	0.0125
Smoking habit	1.886	0.743 – 4.787	0.1818

[a]Cox regression analysis

[b]score of TLR3-t expression: percentage of positive tumor cells ≥3

[c]score of TLR3-s and TLR3-i expression: percentage of positive immune cells ≥1
